# Immobilization of the Highly Active UDP-Glucose Pyrophosphorylase From *Thermocrispum agreste* Provides a Highly Efficient Biocatalyst for the Production of UDP-Glucose

**DOI:** 10.3389/fbioe.2020.00740

**Published:** 2020-07-02

**Authors:** Antje Kumpf, Daria Kowalczykiewicz, Katarzyna Szymańska, Maria Mehnert, Isabel Bento, Aleksandra Łochowicz, André Pollender, Andrzej Jarzȩbski, Dirk Tischler

**Affiliations:** ^1^Institute of Biosciences, Environmental Microbiology, TU Bergakademie Freiberg, Freiberg, Germany; ^2^Department of Microbial Biotechnology, Faculty of Biology and Biotechnology, Ruhr-Universität Bochum, Bochum, Germany; ^3^EMBL Hamburg, Hamburg, Germany; ^4^Department of Organic Chemistry, Bioorganic Chemistry and Biotechnology, Silesian University of Technology, Gliwice, Poland; ^5^Biotechnology Centre, Silesian University of Technology, Gliwice, Poland; ^6^Department of Chemical Engineering and Process Design, Silesian University of Technology, Gliwice, Poland; ^7^Institute of Chemical Engineering, Polish Academy of Sciences, Gliwice, Poland

**Keywords:** *Thermocrispum agreste* DSM 44070, NDP-sugars, UTP, immobilization, silica carrier, mesostructured cellular foams, MCF

## Abstract

Biocatalysis that produces economically interesting compounds can be carried out by using free enzymes or microbial cells. However, often the cell metabolism does not allow the overproduction or secretion of activated sugars and thus downstream processing of these sugars is complicated. Here enzyme immobilization comes into focus in order to stabilize the enzyme as well as to make the overall process economically feasible. Besides a robust immobilization method, a highly active and stable enzyme is needed to efficiently produce the product of choice. Herein, we report on the identification, gene expression, biochemical characterization as well as immobilization of the uridine-5′-diphosphate-glucose (UDP-glucose) pyrophosphorylase originating from the thermostable soil actinobacterium *Thermocrispum agreste* DSM 44070 (*Ta*GalU). The enzyme immobilization was performed on organically modified mesostructured cellular foams (MCF) *via* epoxy and amino group to provide a stable and active biocatalyst. The soluble and highly active *Ta*GalU revealed a *V*_max_ of 1698 U mg^–1^ (uridine-5′-triphosphate, UTP) and a *K*_m_ of 0.15 mM (UTP). The optimum reaction temperature was determined to be 50°C. *Ta*GalU was stable at this temperature for up to 30 min with a maximum loss of activity of 65%. Interestingly, immobilized *Ta*GalU was stable at 50°C for at least 120 min without a significant loss of activity, which makes this enzyme an interesting biocatalyst for the production of UDP-glucose.

## Introduction

Uridine-5′-diphosphate-glucose (UDP-glucose) is a fundamentally important molecule in biology, food, biopharmaceuticals and cosmetic chemistry. It is one of the key precursors for sugar interconversion, for formation of di- and polysaccharides, and in amino and nucleotide sugar metabolism. In addition, UDP-glucose can be used as a source for other industrial interesting compounds such as antibiotics (Lai et al., 2008; Rodríguez-Díaz and Yebra, 2011; Gutmann and Nidetzky, 2016; Huang et al., 2016; Schmölzer et al., 2017; Mestrom et al., 2019; Liu et al., 2020). A number of chemical methods for UDP-glucose synthesis have already been proposed (Moffatt and Khorana, 1958; Hanessian et al., 1998), but they create reactivity and selectivity problems, often requiring modification of functional groups to protect those residues of the sugar molecules that should not react, and at the same time expose those groups that should react (Guo and Ye, 2010). Nevertheless, in many cases, the stereoselectivity is not obtained and additional bisacetal, carbonate or xylylene groups have to be used to promote the stereochemical outcome needed for the axial or the equatorial conformation (Guo and Ye, 2010). Increases in the stereoselectivity of more than 90% have been observed for one of the stereoisomer, but under harsh reaction conditions (e.g., temperatures of −78°C or pressures of 3 atm) and with harmful chemicals (e.g., dichloromethane, dioxane, DBU or *tert*-butyldimethylsilyl chloride) (Guo and Ye, 2010). In contrast to this type of chemical synthesis, enzymatic sugar coupling offers several advantages. Enzymatic reactions are carried out under mild reaction conditions (pH, temperature), in the range of atmospheric pressure and often using aqueous solvents (Rasor and Voss, 2001; Schmid et al., 2002; Sheldon and van Pelt, 2013; Rajapriya et al., 2018). Indeed, these processes catalyzed by enzymes are often referred to as “white biotechnology,” which underlines their positive impact on the environment (Grunwald, 2015; Sheldon and Woodley, 2018). Occasionally, when high instability of the enzymes is observed, whole cells can also be used to catalyze these processes (Lin et al., 2013; Rajapriya et al., 2018). Furthermore, application of whole cells, with upregulated genes that encode for the protein(s) of interest and where proteins are protected by the cell envelope (Ni et al., 2006; de Carvalho, 2011), can also be seen as an advantage. Indeed, problems such as the supply of expensive co-factors and harsh conditions with high shearing forces, extreme pHs and wide temperature changes, that may occur during these processes, will be minimized. However, sometimes the cell does not allow overproduction of the protein(s) of interest, in particular, when this interferes with the energy life cycle of the host organism (Brown and Kornberg, 2004; Manganelli, 2007), which compromises the production of larger amounts of protein. In addition, product degradation and/or the synthesis of unnecessary by-products, which are the result of the presence of other enzymes of the cell’s metabolic shock can constitute an even bigger problem (Park et al., 2020). Actually, this can be the case during UDP-glucose biosynthesis by means of a whole cell system, as several pathways will utilize it and limit the amount of product for a subsequent down-stream processing. Furthermore, the down-stream processing needs to be carried out under mild conditions as UDP-glucose is not very stable and decomposes for example at non-neutral pH conditions or in the presence of reactive solvents (Hill et al., 2017).

Considering this, it is of utmost importance that our civilization realizes that treating enzymes as catalytic moieties for industrial application will have a critical impact in mitigating pollution and in the reconversion of valuable resources. The application of relatively expensive catalysts, such as enzymes, imposes the need for using them multiple times, or in a continuous process, making use of their stability and full functional quality under reaction conditions (Liese and Hilterhaus, 2013; Sheldon and van Pelt, 2013). One of the possibilities to fulfill these requirements is enzyme immobilization. Indeed, the application of immobilized enzymes allows for significant simplification of the reactor’s structure and precise control of the process, e.g., stopping it by separating the catalyst from the reaction mixture. Additionally, immobilization increases the stability of the enzyme by multi-point interaction with the carrier’s surface, generates a favorable micro-environment and protects against intermolecular interactions (Mateo et al., 2007; Liese and Hilterhaus, 2013; Rodrigues et al., 2013; Zhang et al., 2015). Therefore, immobilization of enzymes is usually required in industrial applications (Mateo et al., 2007; Zhang et al., 2015). An effective and very common immobilization method is the covalent binding toward carriers. Indeed, strong interaction between the functional groups of the enzyme and the support leads to a high activity of the biocatalyst even after immobilization (Hassan et al., 2019).

For the formation of uridine-5′-diphosphate-glucose (UDP-glucose) several thermostable nucleotide-5′-diphosphate (NDP)-sugar pyrophosphorylases from thermophilic bacteria, like *Thermus caldophilus* (Kim et al., 1999) or *Thermodesulfatator indicus* (Sohn et al., 2006; Li et al., 2017) or thermophilic archaea, like *Sulfolobus tokodaii* (Zhang et al., 2005; Honda et al., 2017), have been reported and characterized. Even NDP-sugar pyrophosphorylases from mesophilic bacteria, like *Helicobacter pylori* (Mizanur and Pohl, 2008), have been found to be active and stable. One group of NDP-sugar pyrophosphorylases are UDP-glucose pyrophosphorylases (EC 2.7.7.9; alternative name: UTP-glucose-1-phosphate uridylyltransferase; abbreviations: GalU, UGPase) that convert α-D-glucose 1-phosphate (G1P) and uridine-5′-triphosphate (UTP) into UDP-glucose and inorganic pyrophosphate (PPi). This group of enzymes is known for years, but it has been only rarely employed as biocatalyst to produce UDP-glucose. Indeed, at industrial level, sucrose synthases (SuSy) have been used, instead (Gutmann and Nidetzky, 2016; Schmölzer et al., 2017). SuSy representatives are stable and can be employed as whole cell biocatalysts (Schmölzer et al., 2017), but the reaction toward UDP-glucose is not favored with respect to the equilibrium at certain pH-ranges and lower UDP concentrations (Gutmann and Nidetzky, 2016), and the enzymes show activities in a range of 14–53 μmol min^–1^ mg^–1^. The kinetic drawbacks of SuSy enzymes are not present when considering UDP-glucose formation by GalU representatives. Indeed, for GalUs lower substrate (UTP) concentrations can be applied and still obtain activities of 0.12 and up to 5⋅10^6^ μmol min^–1^ mg^–1^ (Gustafson and Gander, 1972; Steiner et al., 2007; Lai et al., 2008; Asención Diez et al., 2015; Ebrecht et al., 2015; Zavala et al., 2017). Furthermore, our recent work on UDP-glucose pyrophosphorylases demonstrated that actinobacteria such as rhodococci harbor also highly active GalU representatives (Kumpf et al., 2019). These actinobacterial GalUs can be promising alternatives to produce UDP-glucose. However, these enzymes seem to be rather unstable. Therefore, using *in silico* tools, we have screened for related actinobacteria that are thermophilic and encode for enzymes with a high similarity on amino acid level. Our objective was to find a highly active and stable biocatalyst suitable for applied studies.

Herein, we report the biochemical characterization of the first thermostable and highly active UDP-glucose pyrophosphorylase from the thermophilic soil actinobacterium *Thermocrispum agreste* DSM 44070 (*Ta*GalU). Furthermore, and in order to obtain a stable and easy to use heterogeneous catalyst, *Ta*GalU was covalently immobilized on organically modified mesostructured cellular foams (MCF) functionalized with either amino or epoxy groups and further characterized. Indeed, silica is environmentally acceptable, structurally more stable and more resistant to microbial attacks. The respective monoliths carrying the loaded enzyme have a surface, which can be densely covered with various anchor groups. Due to the large area and the presence of pores with diameters larger than those of enzyme molecules, a considerable surface area can be activated.

## Materials and Methods

### Bacterial Strains, Plasmids and Gene Synthesis

Table 1 shows all strains, plasmids and primers that were used in this study.

**TABLE 1:** Strains, plasmids and primers.

Strain, plasmid, gene or primer	Relevant characteristics	Source or reference
*E. coli* DH5α	fhuA2 Δ(argF-lacZ)U169 phoA glnV44 Φ80 Δ(lacZ)M15 gyrA96 recA1 relA1 endA1 thi-1 hsdR17	New England Biolabs Inc.
*E. coli* BL21(DE3) pLysS	fhuA2 [lon] ompT gal (λ DE3) [dcm] ΔhsdS λ DE3 = λ sBamHIo ΔEcoRI-B int::(lacI::PlacUV5::T7 gene1) i21 Δnin5	New England Biolabs Inc.
pET16bP	pET16b with additional multiple cloning site; allows production of recombinant proteins with *N*-terminal Histidine_10_-Tag and gene expression induction with IPTG	Tischler et al., 2009
pET16bP-*TagalU*	pET16bP vector with recombinant UDP-glucose-pyrophosphorylase gene of *T. agreste* DSM 44070 (*TagalU*)	Eurofins Genomics
pET16bP-fw	5′-CATCACAGCAGCG GCCATATCGAAG-3′	This study
pET16bP-rev	5′-CAGCTTCTTTTC GGGCTTTGTTAG-3′	This study

The pET16bP-*TagalU* plasmid used in this study was synthesized by Eurofins Genomics (Ebersberg, Germany). It consisted of a backbone (5740 bp) coding for a resistance against Ampicillin and the codon usage optimized UDP-glucose pyrophosphorylase (*galU*) gene from *Thermocrispum agreste* DSM 44070 as insert (911 bp, NCBI accession of the protein sequence: WP_028847555; GenBank accession of the codon usage optimized nucleotide sequence: MT321102; see Supplementary Material) under the control of the lactose or isopropyl β-D-1-thiogalactopyranoside (IPTG) inducible lac-promoter. The inserted *galU* sequence is flanked by the restriction sites of NdeI and NotI. In addition, the pET16bP backbone contained a DNA sequence that allowed the production of the GalU protein with a *N*-terminal 10x histidine-tag.

### Transformation, Protein Production and Purification

Transformation of *E. coli* BL21(DE3) pLysS was carried out as recommended by New England Biolabs Inc. (Ipswich, MA, United States).

Protein production was performed in a 10-liter fermenter with TB autoinduction medium (Studier, 2005) with 100 mg L^–1^ ampicillin as well as 50 mg L^–1^ chloramphenicol. The expression culture was inoculated 1:50 with an overnight pre-culture of *E. coli* BL21(DE3) pLysS-pET16bP-*TagalU* in the same medium used for the expression culture. The main culture was incubated at 37°C until an OD_600_ of 2.0–2.5 was observed, followed by temperature reduction to 20°C and gene expression with respective protein production for 22 h.

After 22 h cells were harvested and centrifuged at 4°C, 5,000 × g for 30 min. Pelleted cells were resuspended with ca. 20 mL of a FPLC equilibration buffer (25 mM sodium phosphate buffer, pH 7.1, 300 mM sodium chloride) and frozen at −80°C.

For purification, the cell suspensions were thawed as fast as possible in a warm water bath and the following solutions were added per portion: 1 mM magnesium chloride (MgCl_2_), 240 U DNase I and 12 mg Lysozyme. If necessary 5–10 mL of FPLC equilibration buffer was added, in case the suspension was too viscous. The suspension obtained was mixed and incubated for 30–60 min at 30°C. Cells were disrupted by 10 cycles of sonification for 30 s at an intensity of 70% (Bandelin SONOPLUS sonifier) and cooling steps on wet ice. Crude cell extract was centrifuged at 12,000 × *g* at 4°C for 20 min. The pellet obtained had the insoluble protein fraction (IP) comprising inclusion bodies and remaining cell debris. Another two centrifugation steps with the supernatant were carried out at 50,000 × g at 4°C for 30–45 min each. The clear supernatant was filtered through a 0.45 μm and a 0.22 μm filter. The fraction obtained was the soluble protein fraction (SP) used for subsequent experiments. The prepared SP fraction was loaded on a GE Healthcare HisTrap HP 5 mL nickel column that was pre-equilibrated with FPLC equilibration buffer. Protein loading was performed with equilibration buffer and 25 mM imidazole. After washing with equilibration buffer and 40 mM imidazole and application of a linear gradient from 40 to 500 mM imidazole, the *Ta*GalU was eluted at 500 mM imidazole.

Fractions that showed UDP-glucose pyrophosphorylase activity were pooled together and the protein was precipitated with 80% saturated ammonium sulfate [(NH_4_)_2_SO_4_] solution. After gentle mixing and centrifugation at 12,000 × g at 4°C for 45 min the supernatant was discarded, and the pelleted protein was stored at 4°C. For experimentation, (NH_4_)_2_SO_4_ precipitated *Ta*GalU was dissolved and diluted to the appropriate concentration in 50 mM Hepes, pH 7.0, 100 mM sodium chloride, 1 mM MgCl_2_ and stored for up to 2 weeks at 4°C without significant loss of activity.

### Protein Determination

Protein concentration was determined by measuring the absorption signal at 280 nm with a NanoDrop using the theoretical extinction coefficient (11,585 cm^–1^ M^–1^) and the theoretical molecular weight (33,678 Da) calculated from the amino acid sequence by means of ExPASy ProtParam online tool. The detection of the molecular weight of the purified *Ta*GalU was done by SDS-PAGE and Coomassie staining (Laemmli, 1970) under denaturizing conditions. The oligomeric state of the *Ta*GalU was monitored under non-denaturizing conditions by size exclusion chromatography using a 24 mL Superdex 200 10/300 GL column (GE Healthcare) and a calibration standard mix containing ferritin, conalbumin, carbonic anhydrase, ribonuclease and aprotinin in 50 mM Hepes buffer pH 7.0 and 1 mM MgCl_2_. The void volume of the column was measured with dextran blue.

### Standard Enzyme Activity Assay for *Ta*GalU

The enzyme activity was only measured in the direction of UDP-glucose formation in a reaction volume of 1 mL for the free enzyme and of 3 mL for the immobilized enzyme, with a standard test of a buffer containing 2 mM UTP, 2 mM G1P, 4 mM MgCl_2_, 50 mM Hepes, pH 7.0 and an appropriate amount of *Ta*GalU, if not otherwise indicated. Pre-incubation of the reaction mixture was carried out at 50°C for 15 min, enzyme was added after storage on ice to start the reaction. After defined time points, 100 μL samples were taken and the reaction was stopped by adding 100 μL acetonitrile followed by vortexing and centrifugation at 20,000 × g for 2 min. Hundred μL of the clear supernatant was used for HPLC separation combined with UV/Vis-detection of the product UDP-glucose (see section “HPLC Measurement and Determination of the Specific *Ta*GalU Enzyme Activity”).

In case of the immobilized enzyme, the reaction was stopped by filtering off the carrier with biocatalyst. The filtered samples (100 μL) were diluted with 100 μL acetonitrile, vortexed and analyzed by HPLC (see section “HPLC Measurement and Determination of the Specific *Ta*GalU Enzyme Activity”).

To increase the activity to a maximum, the reaction conditions were adjusted to optimal conditions systematically during experimentation.

### HPLC Measurement and Determination of the Specific *Ta*GalU Enzyme Activity

HPLC detection of the product UDP-glucose for the free enzyme was performed with a Thermo Scientific Dionex Ultimate 3000 UHPLC with UV/Vis detection and a Macherey-Nagel EC 150/4.6 Nucleoshell HILIC column with 2.7 μm particle size. The product formation for the immobilized *Ta*GalU was measured with an Agilent Technologies 1200 Series HPLC equipped with UV/Vis detector and a Thermo Scientific Accucore-150-Amide-HILIC Column (100 × 3 mm) with 2.6 μm particle size. UDP-glucose formation was monitored at a wavelength of 260 nm with an isocratic method with 70 % acetonitrile and 30 % ammonium acetate buffer (134 mM, pH 5.35). A flow rate of 0.8 mL min^–1^ and 3 μL of injected sample was used for the Thermo Scientific column and a flow rate of 1.3 mL min^–1^ and 5 μL injection volume for the Macherey-Nagel column. The column temperature was set to 30°C for both systems. Calibration was carried out with UDP-glucose standards of appropriate concentrations mixed with acetonitrile, to have the same concentration as in samples from enzyme assays (50%-vol each sample and acetonitrile). The peak area generated by UDP-glucose was used for data evaluation and referred to the time points of the enzyme assay to calculate the product formation and initial product formation rates, respectively.

The activities were plotted according to enzyme kinetic models for analysis, by using the following equations according to Michaelis–Menten (1) for non-limiting conditions and Yano and Koga (1969) (2) for substrate inhibition:

(1) $$V=\frac{v_{\max}\cdot c_{S}}{K_{m}+c_{S}}$$

(2) $$V=\frac{v_{\max}\cdot c_{S}}{K_{m}+c_{S}+\frac{c_{S}^{3}}{K_{i}^{2}}}$$

Initial values were obtained as product amount per time and were converted to enzyme units (U). 1 U is defined as 1 μmol_UDP–glucose_ formed per minute. This was used to calculate (specific) activities (*V* or *V*_max_) in U mg^–1^ (μmol_UDP–glucose_ min^–1^ mg^–1^_enzyme_) and rates are given as turnover frequencies or *k*_cat_ in s^–1^ (μmol_UDP–glucose_ μmol^–1^_enzyme_ s^–1^).

### Preparation and Functionalization of Siliceous Carriers

The MCFs were prepared as reported before (Szymańska et al., 2009; Bryjak et al., 2012). Functionalization with amino and epoxy groups was done by gently stirring of 1 g of dry MCF with either 270 μL of 3-aminopropyltrimetoxysilane or 340 μL 3-glycidoxypropyltrimetoxysilane, depending on the attached groups, in 25 mL of dry toluene at 85°C for 24 h and subsequent drying on air.

### Immobilization of *Ta*GalU on Carriers With Amino and Epoxy Functionalization

Preliminary to immobilization 400 mg of carriers were washed with 15 mL ethanol (1x), distilled water (2x) and 100 mM phosphate buffer, pH 7.0 (2x). After each step, the samples were centrifuged at room temperature and 8,667 × g for 20 min. The amino groups were activated with glutaraldehyde (GA) by incubating the carriers shaking with 30 mL of 2.5% (v/v) glutaraldehyde solution in 100 mM phosphate buffer pH 7.0 for 1.5 h. The unbound GA was eluted with distilled water and 100 mM phosphate buffer pH 7.0, before adding 16 mL of *Ta*GalU with a concentration of 1.45 mg mL^–1^ in 100 mM phosphate buffer, pH 5.5, 6.0, 6.8, 7.6. The suspension was mixed gently for 3 h at room temperature and stored at 4°C overnight.

After overnight incubation, the carriers were centrifuged at 8,667 × *g* and 5°C for 20 min. Unbound protein was removed by washing with 100 mM phosphate buffer pH 7.0 (1x), 100 mM phosphate buffer pH 7.0 with 500 mM NaCl (2x), 100 mM acetate buffer pH 4.5 (1x) and several times with distilled water to remove the acid. The supernatants of each washing step were kept to determine the amount of immobilized protein. To block unreacted active groups, the carriers were suspended in 500 mM Tris-HCl buffer, pH 7.8 overnight at 4°C. Before the enzyme assays were carried out, the carriers were centrifuged (8,667 × *g*, 20 min, 5°C) and washed with 100 mM phosphate buffer pH 7.0. The amount of immobilized enzyme was determined by measuring the amount of unbound protein in the washing step solutions by spectrophotometry at 280 nm or by Lowry method using bovine serum albumin as a standard (Lowry et al., 1951) and subtracting it from the amount of the total applied protein.

## Results

### Recombinant Expression of *TagalU* in *E. coli*, Protein Production, Purification and Identification

**FIGURE 1:**
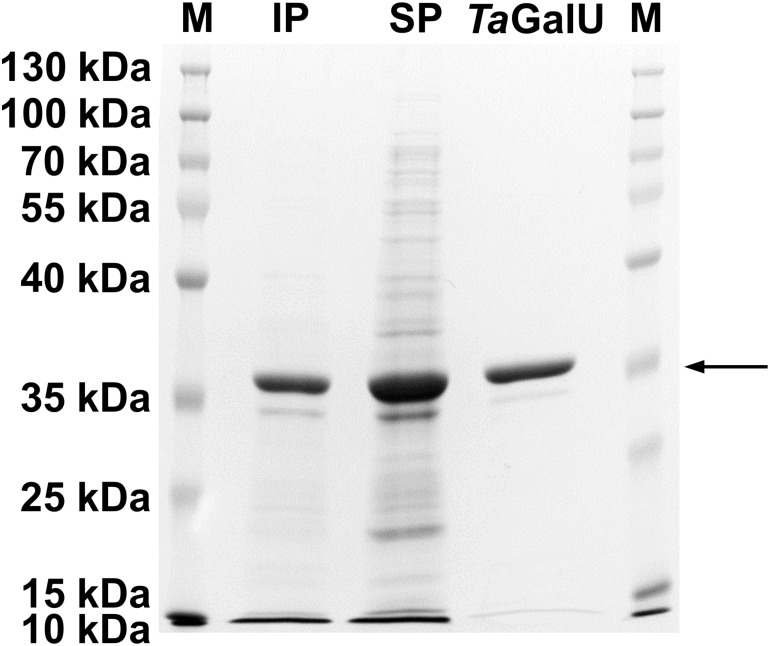
SDS-PAGE gel of *Ta*GalU after purification. The arrow indicates the expected molecular weight of 33.7 kDa. M, protein marker (Thermo Scientific^TM^ PageRuler^TM^ Prestained Protein Ladder); IP, insoluble protein fraction; SP, soluble protein fraction.

After successful transformation of *E. coli* BL21(DE3) pLysS cells with the pET16bP-*TagalU* plasmid, the recombinant enzyme *Ta*GalU was produced with a maximum yield of 73 mg of purified protein per liter of broth. Figure 1 shows the occurrence of the protein in both fractions, the soluble and the insoluble one, indicating that functional protein and inclusion bodies were formed. The expected molecular weight of 33.7 kDa was verified.

**FIGURE 2:**
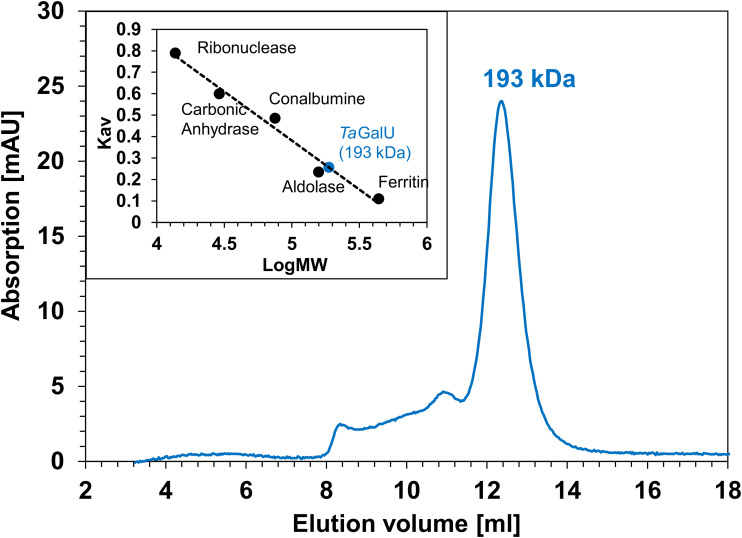
Size exclusion chromatography of *Ta*GalU. Elution chromatogram of *Ta*GalU during size exclusion and calibration curve established with the calibration standards ribonuclease, carbonic anhydrase, conalbumin, aldolase and ferritin are shown.

Size exclusion chromatography determined the oligomeric state of *Ta*GalU to be a hexamer of about 193 kDa (see Figure 2).

### Finding Optimal Reaction Conditions for the Free *Ta*GalU

To figure out the most useful reaction conditions, initial experiments were carried out only with the free enzyme and by varying the reaction components of the standard enzyme test. At first, the reaction temperature and the protein concentration per reaction were tested. The temperature and the protein concentration were increased as follows: 10, 22, 30, 50, and 80°C; 0.013 μg mL^–1^, 0.04 μg mL^–1^, 0.08 μg mL^–1^, and 8 μg mL^–1^, respectively. Assays containing less than 0.013 μg mL^–1^ of protein did not yield analyzable results, as the amount of product was too low at the beginning of the reaction. Details on the reaction optimization are presented in Table 2. The highest specific activity of 1861 U mg^–1^ was observed with 0.013 μg mL^–1^ at 50°C, so these conditions were used for further experiments. Reaction solutions contained the same components like in the standard enzyme activity test in section “Materials and Methods” in 1 mL scale.

**TABLE 2:** Initial tested conditions for the *Ta*GalU reaction and comparison of observed enzyme activities.

Temperature	*Ta*GalU concentration	Observed *Ta*GalU activity
10°C	0.013 μg ml^–1^	213 U mg^–1^
	0.04 μg ml^–1^	42.4 U mg^–1^
	0.08 μg ml^–1^	15.6 U mg^–1^
	8 μg ml^–1^	11.2 U mg^–1^
22°C	8 μg ml^–1^	61.7 U mg^–1^
30°C	8 μg ml^–1^	68.4 U mg^–1^
50°C	**0.013 μg ml^–1^**	**1861 U mg^–1^**
	0.04 μg ml^–1^	395 U mg^–1^
	0.08 μg ml^–1^	157 U mg^–1^
80°C	0.013 μg ml^–1^	n.d.
	0.04 μg ml^–1^	n.d.
	0.08 μg ml^–1^	n.d.

*Reaction solutions contained 2 mM UTP, 2 mM G1P, 4 mM MgCl_*2*_, 50 mM Hepes, pH 7.0, indicated amounts of *Ta*GalU and reaction was carried out in 1 ml at mentioned temperatures. Best conditions and highest specific activity is written in bold. n.d., not detectable.*

The effect of magnesium ions on the free enzyme was monitored by varying the MgCl_2_ concentration between 0 and 100 mM using the standard activity assay (see Supplementary Figure 1). As the highest relative activity could be observed with 3 mM MgCl_2_, which corresponded to a specific activity of 2111 U mg^–1^, this concentration was used for the most subsequent experiments.

To test the cofactor specificity, we also used manganese chloride (MnCl_2_), nickel chloride (NiCl_2_), cobalt chloride (CoCl_2_), calcium chloride (CaCl_2_) and zinc chloride (ZnCl_2_) instead of MgCl_2_ with an improved activity assay with 2 mM UTP and 30 mM G1P. Initial experiments with different substrate concentrations could show an increased specific activity for those substrate concentrations. As the protein is stored at 4°C as (NH_4_)_2_SO_4_-pellet without addition of MgCl_2_, *Ta*GalU was dissolved and diluted for this experiment only with 50 mM Hepes, pH 7.0 and 100 mM NaCl. We also tested MgSO_4_, as it was shown with the related *Ro*GalU2 from *Rhodococcus opacus* 1CP that the activity was increased by 40% (Kumpf et al., 2019). Five of seven of the used metal salts can be used for the *Ta*GalU reaction with at least 65% of remaining activity (see Supplementary Figure 2). Only in presence of NiCl_2_ and CaCl_2_, no *Ta*GalU activity was detectable. In this course, also the application of MgSO_4_ increased the *Ta*GalU activity by about 15%.

In addition, the following buffers where tested for the enzyme reaction at improved conditions with 2 mM UTP and 30 mM G1P in 50 mM of respective buffer at pH 7.0: Hepes, Bis-Tris, Mops, sodium phosphate, imidazole as well as Tris-HCl (see Supplementary Figure 3). The choice of the buffer does not have a strong influence on the activity of *Ta*GalU, as the tested buffers only decreased the relative activity by a maximum of 20% when compared to Hepes buffer. Since, we already chose the best buffer, we kept 50 mM Hepes at pH 7.0.

The inactivation properties of the free enzyme were also tested by adding up to 1 M of solvent or inhibitor to the reaction mixture of the improved test with 2 mM UTP, 30 mM G1P, 3 mM MgCl_2_, 50 mM Hepes, pH 7.0 and 50°C. After 30 min, the reactions were stopped by addition of equal amounts of acetonitrile. The following solvents/inhibitors were tested: 2-mercaptoethanol, dimethyl sulfoxide (DMSO), acetonitrile, methanol, ethanol, isopropanol, 1 M each, and 1 mM and 50 mM ethylenediaminetetraacetic acid (EDTA), respectively (see Supplementary Figure 4). The conversion of UTP into UDP-glucose without inhibitor or solvent was 75.5%. Even with the addition of 1 M of each solvent or inhibitor the conversion decreased max. by 6.8% to a residual conversion of 68.7%. In addition, 1 mM EDTA was not sufficient enough to complex MgCl_2_ to inactivate the enzyme. Only the addition of 50 mM EDTA decreased the activity almost completely to 1% residual conversion.

### Immobilization of *Ta*GalU on MCF

Covalent binding of enzymes toward a carrier surface is one of the most stable and effective methods for enzyme immobilization and is based on the covalent attachment of enzymes to water insoluble matrices. Two main parameters can be specified that influence the enzyme activity and stability after immobilization: the type of the functional group of the carrier and the immobilization pH. Therefore, in this work MCF with two types of functional groups (amino: A, and epoxy: E) were tested in the pH range between 5.3 and 7.8 (Jarzȩbski et al., 2007; Szymańska et al., 2007; Szymańska et al., 2013). The amount of protein successfully linked to the MCF carrier was determined as followed (functional group/pH) in relation to the applied protein: A/5.5 (48%), A/6.0 (78%), A/6.7 (43.8%), A/7.8 (66%), E/5.3 (100%), E/6.0 (100%), E/6.7 (86.3%), and E/7.8 (82.3%) (relative values are given according to the method described in section “Immobilization of *Ta*GalU on Carriers With Amino and Epoxy Functionalization”).

**FIGURE 3:**
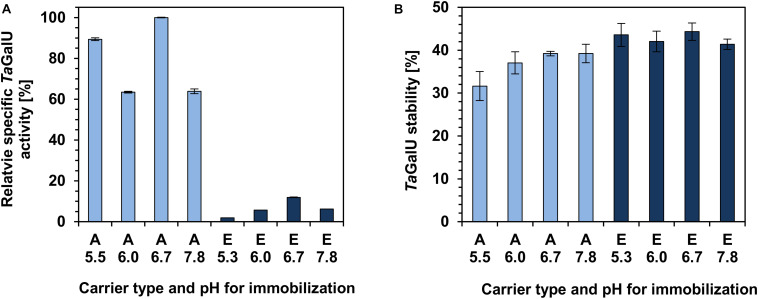
The effect of functional groups of carriers (A, amino; E, epoxy) and immobilization pH on the specific activity **(A)** and storage stability **(B)** of *Ta*GalU. Reaction conditions: 18 μg mL^– 1^ *Ta*GalU immobilized on MCF, 2 mM UTP, 2 mM G1P, 50 mM Hepes buffer, pH 7.0, 4 mM MgCl_2_, 30°C in 3 mL reaction volume and 5 min reaction time. Storage stability was measured with the same setup but after 1 month of storage at 4°C. **(A)** The highest activity of this experiment was set to 100%. **(B)** The initial activities were set to 100%. Means with standard deviations of duplicate measurements are shown.

It was observed that the relative specific activity of *Ta*GalU immobilized on amino functionalized silica is at least five times higher compared to the activity of *Ta*GalU immobilized on epoxy-functionalized silica. The highest specific activity was measured with *Ta*GalU immobilized on amino modified MCF at pH 6.7 (see Figure 3A). After 1 month of storage all biocatalyst preparations obtained showed only about 40% of their initial activity (see Figure 3B). Considering both, the activity and stability, further experiments were carried out using amino-modified MCF, and an immobilization pH of 6.7.

**FIGURE 4:**
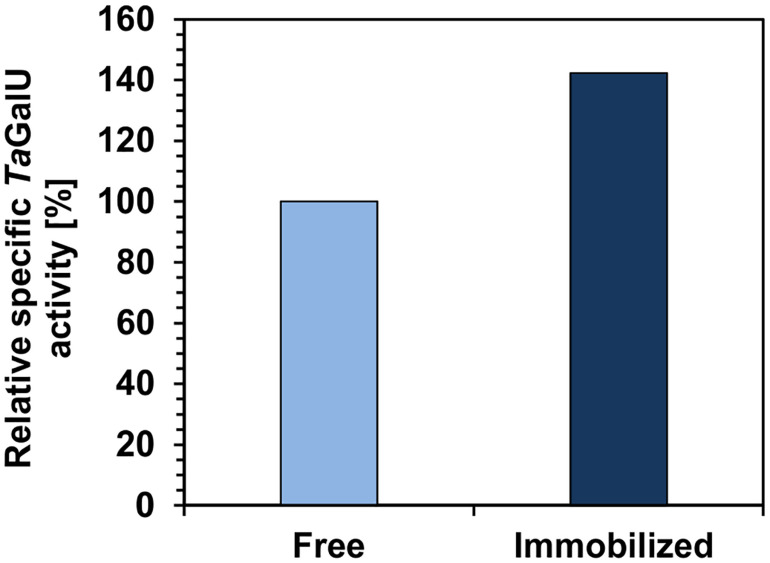
Relative specific activities of free *Ta*GalU and immobilized *Ta*GalU on MCF. Reaction solution contained 2 mM UTP, 2 mM G1P, 3 mM MgCl_2_, 50 mM Hepes buffer, pH 7.0, 0.3106 μg mL^– 1^ *Ta*GalU. Reaction was carried out at 50°C in 3 mL reaction volume. Free *Ta*GalU activity (initial: 1016 U mg^–1^ and after 1.5 years of storage at 4°C: 66 U mg^–1^) was set to 100% (66 U mg^–1^). Enzyme activity of *Ta*GalU was measured directly before and after immobilization considering the determined amount of enzyme in each assay. Immobilized enzyme used herein was linked to the MCF carrier *via* amino group at pH 6.7.

To deeply illustrate the impact of immobilization on enzyme activity, the specific activity of *Ta*GalU before and after immobilization on amino modified MCF at pH 6.7 was measured (Figure 4). The activity tests were done using the same procedure and enzyme from the same batch. It can be seen that the specific activity of immobilized *Ta*GalU increased by 40% in relation to free enzyme. However, the enzyme batch that was used for the immobilization for this experiment was already stored for 1.5 years at 4°C. The remaining specific activity of this enzyme was 66.03 U mg^–1^ and demonstrates how stable this enzyme is even without immobilization.

### Comparison of the Performances of the Free *Ta*GalU With the Immobilized Enzyme

**FIGURE 5:**
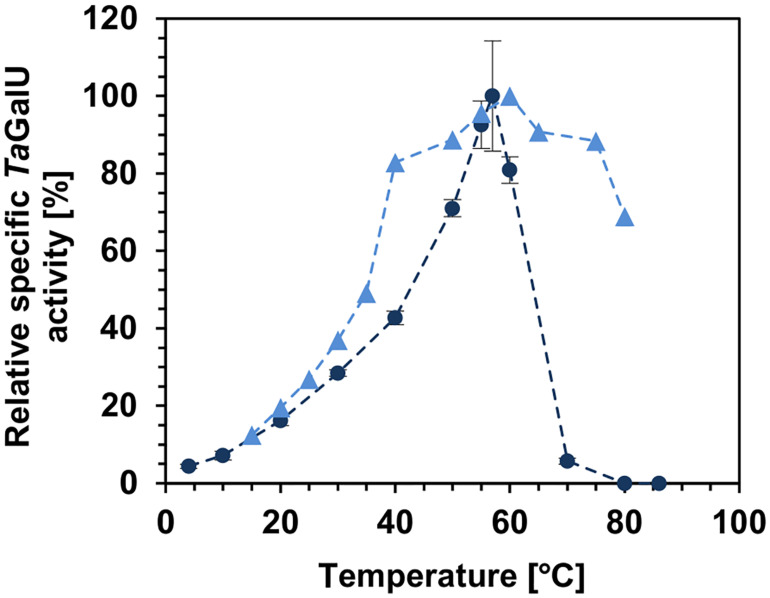
Relative specific *Ta*GalU activity of the free and immobilized enzyme depending on the reaction temperature. Free enzyme (dark blue dots): Reaction conditions are 2 mM UTP, 2 mM G1P, 4 mM MgCl_2_, 50 mM Hepes buffer, pH 7.0, 0.013 μg mL^–1^ free *Ta*GalU, 0–86°C in 1 mL reaction volume. 100% relative specific activity corresponds to 1430 U mg^–1^. Means with standard deviations of triplicate measurements are shown. Immobilized enzyme (blue triangles): Reaction conditions are 2 mM UTP, 2 mM G1P, 4 mM MgCl_2_, 50 mM Hepes buffer, pH 7.0, 0.3109 μg mL^–1^ *Ta*GalU immobilized amino modified MCF, 15–80°C in 3 mL reaction volume. No replicates for immobilized enzyme presented, but were performed for 30°C, 40° and 50° with a standard deviation lower than 5%. The highest activities of free and immobilized *Ta*GalU were set as 100%.

As the optimal reaction and immobilization conditions for *Ta*GalU were established, a deeper look inside the performance of the free and immobilized enzyme was made. At first, the enzyme activity at different temperatures was investigated. To monitor the effect on the specific activity a temperature profile was done from 0 to 85°C for the free enzyme and from 15 to 80°C for the immobilized *Ta*GalU, respectively (Figure 5). The maximum activity of the free enzyme and for the immobilized variant were measured at 57°C and at 60°C, respectively (see Figure 5). However, further temperature increase resulted in a significant decrease of the activity of the free enzyme, whereas immobilized *Ta*GalU still showed higher activities. At 60°C the relative activity of the free enzyme was 80% of its maximum activity, and when the reaction temperature increased further to 70-80°C, it completely lost its activity. The immobilized *Ta*GalU showed 80 and 60% of its maximum activity at 70 and 80°C, respectively. In addition, the temperature region where the activity surpassed 60% of its maximum is broader for the immobilized *Ta*GalU, from 40 to 80°C, whereas for the free enzyme it was only from 50 to 60°C. It was shown that 50°C (relative activity 71% = 1015 U mg^–1^ with respect to maximum turnover) is an optimum temperature for further experimentation for both the free and the immobilized enzyme.

**FIGURE 6:**
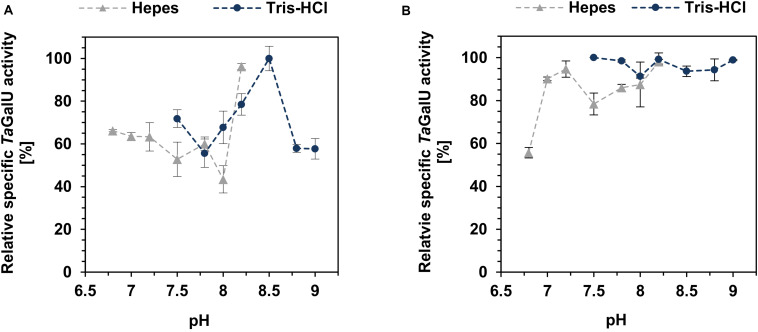
Effect of pH on the free *Ta*GalU **(A)** and the immobilized catalyst **(B)**. **(A)** Reaction solution contained 2 mM UTP, 2 mM G1P, 3 mM MgCl_2_, 50 mM buffer, pH 6.8 – 9.0, 0.013 μg mL^–1^ free *Ta*GalU, 50°C in 1 mL reaction volume. 100% relative specific activity corresponds to 2876 U mg^–1^. Means with standard deviations for triplicate measurements are shown. **(B)** Reaction conditions are 2 mM UTP, 2 mM G1P, 3 mM MgCl_2_, 50 mM buffer, pH 6.8 – 9.0, 0.2591 μg mL^–1^ *Ta*GalU immobilized amino modified MCF. Reaction was carried out at 50°C in 3 mL reaction volume. Means with standard deviations are shown from triplicate reactions. 100% relative specific activity corresponds to 1568 U mg^–1^.

The pH activity of both *Ta*GalU variants was also tested by altering the pH of the Hepes buffer from 6.8 to 8.2 and Tris-HCl buffer from 7.5 to 9 (Figure 6). The free enzyme has a clear preference for higher pH-values with a maximum at pH 8.2. Interestingly, when we compared the pH activity of the free *Ta*GalU with the immobilized enzyme, the activity of the immobilized enzymes was higher on a wider range of pH-values (>90% relative activity in Tris-HCl and >80% relative activity in Hepes buffer) in both buffers between pH 7 and 9. Variation of the pH from 6.8 to 9.0 (6.8 – 8.2: Hepes and 7.5 - 9.0: Tris-HCl) showed, in general, that *Ta*GalU is active over a wide pH range, where the relative activity barely falls below 60%. The highest activity was observed with Tris-HCl at pH 8.5, and corresponded to a specific activity of 2876 U mg^–1^ (Figure 6). Unfortunately, we discovered that even though *Ta*GalU showed the highest activity in Tris-HCl pH 8.5, the protein is too unstable to get reasonable results. Therefore, we decided to stick to Hepes buffer pH 7.0.

**FIGURE 7:**
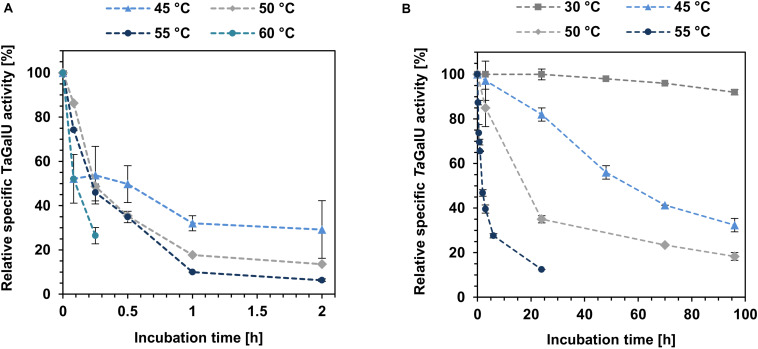
Temperature stability of free **(A)** and immobilized *Ta*GalU **(B)**. **(A)** The reaction solution contained the following ingredients: 2 mM UTP, 30 mM G1P, 3 mM MgCl_2_, 50 mM Hepes, pH7.0, 0.08 μg mL^–1^ *Ta*GalU in 1 mL reaction volume. Incubation and performance of the reaction was carried out at indicated temperatures. 100% relative activity corresponds to the specific activity before incubation. Means of triplicates with standard deviations are shown. **(B)** Reaction solution contained 2 mM UTP, 2 mM G1P, 3 mM MgCl_2_, 50 mM Hepes buffer, pH 7.0, 0.2591 μg mL^–1^ *Ta*GalU immobilized on amino modified MCF. Reaction was carried out at indicated temperatures in 3 mL reaction volume. 100% relative activity corresponds to the specific activity before incubation. Means of triplicates with standard deviations are shown.

One of the most important factors for biocatalysts is their long-term stability under process conditions, particularly, for enzymes as catalysts, reaction temperature should be taken into account. For this reason, free and immobilized *Ta*GalU were stored at different temperatures (30, 45, 50, 55, 60°C) for 2 h (free *Ta*GalU) and 96 h (immobilized *Ta*GalU), respectively, and samples were taken over time to measure the activity (see Figure 7).

For the free *Ta*GalU the initial activity at 60°C is the highest observed with 1873 U mg^–1^ (almost doubled compared to the standard activity value), but it decreases very fast. After 10 min, the formation of protein precipitate was observed. This precipitated protein was not active anymore. The initial activities of the lower temperatures tested were only around 700 U mg^–1^. After 2 h only activities between 50 and 200 U mg^–1^ (about 6 and 29% of the activities before incubation) were observed.

In contrast to the free *Ta*GalU, the immobilized variant is much more stable. After 96 h at 30°C it lost only 4% of its initial activity. At higher temperatures, the activity decreased faster, but was not comparable to the activity decrease of the free enzyme. Hence, the immobilized *Ta*GalU still has 47% of the initial activity after 2 h at 55°C. The free enzyme had only about 6% of the initial activity after 2 h at the same temperature.

To calculate the standard kinetic parameters *K*_m_, *V*_max_, *k*_cat_, *k*_cat_/*K*_m_, two series of experiments were conducted. During the first series, the concentration of G1P was fixed at 30 mM, whereas the concentration of UTP was varied between 0.05 and 6 mM. Next, the concentration of G1P was changed between 0.01 and 45 mM, while the UTP concentration was fixed at 2 mM. The same conditions were applied for the immobilized *Ta*GalU, as well.

**FIGURE 8:**
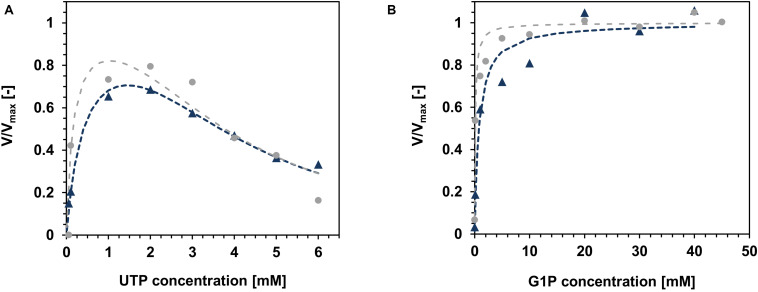
Variation of the substrate concentrations for kinetic modeling. **(A)**: UTP. Reaction mixtures contained 0.05–6 mM UTP, 30 mM G1P, 3 mM MgCl_2_, 50 mM Hepes, pH 7.0, 0.013 μg mL^–1^ free *Ta*GalU and 0.1036 μg mL^–1^ *Ta*GalU on MCF, respectively, reaction temperature was set to 50°C in 1 mL (free *Ta*GalU) and 3 mL (*Ta*GalU on MCF) reaction volume. Data were fitted to the model of Yano and Koga (Yano and Koga, 1969) for substrate inhibition [see section “HPLC Measurement and Determination of the Specific *Ta*GalU Enzyme Activity,” equation (2)] and **(B)**: G1P. Reaction mixture contained 2 mM UTP, 0.01-40 mM G1P (*Ta*GalU on MCF) or 0.01–45 mM G1P (free *Ta*GalU), 3 mM MgCl_2_, 50 mM Hepes, pH 7.0, 0.013 μg mL^–1^ free *Ta*GalU and 0.3109 μg mL ^1^ *Ta*GalU on MCF, respectively, reaction temperature was set to 50°C in 1 mL (free *Ta*GalU) and 3 mL (*Ta*GalU on MCF) reaction volume. Data were fitted to the Michaelis-Menten Equation [section “HPLC Measurement and Determination of the Specific *Ta*GalU Enzyme Activity,” equation (1)]. Gray circles and gray dashed lines show the data points and fits for the free *Ta*GalU, blue triangles and blue dotted lines show the data points and fits for the immobilized *Ta*GalU on MCF.

Both variants of *Ta*GalU showed a strong substrate inhibition at concentrations above 2 mM UTP (Figure 8A), whereas changes in the G1P concentration (Figure 8B) did not show any inhibition and were fitted to the Michaelis–Menten model. The data of UTP were fitted to the Yano and Koga model (Yano and Koga, 1969). With these fits, the calculation of the kinetic constants for the free enzyme was possible and are presented in Table 3. We also calculated the kinetic constants for the immobilized enzyme (see Supplementary Table 1). The values for the inhibition constant *K*_I_ are comparable between both forms of catalysts, whereas the values of the Michaelis constant *K*_m_ are 2.5 times and 6.5 times higher for the immobilized enzyme, pointing out that the affinity of the immobilized *Ta*GalU is lower toward its substrates compared to the free enzyme. This can be ascribed to a hindered access of substrates into proteins embedded in a porous structure of MCFs. As for the immobilization for this experiment an older batch of enzyme with a much lower activity was used, the values for the maximum velocity *V*_max_ and turnover frequency *k*_cat_, as well as the catalytic efficiency *k*_cat_/*K*_m_ are hardly comparable to each other (see Supplementary Table 1).

**TABLE 3:** Kinetic constants for free *Ta*GalU calculated by the fits.

Kinetic constant	UTP	G1P
*K*_m_ [mM]	0.15	0.44
*V*_max_ [U mg^–1^]	1698	1111
*k*_cat_ [s^–1^]	914	598
*K*_I_ [mM]	3.9	–
*k*_cat_/*K*_m_ [μM^–1^ s^–1^]	6.09	1.36

**FIGURE 9:**
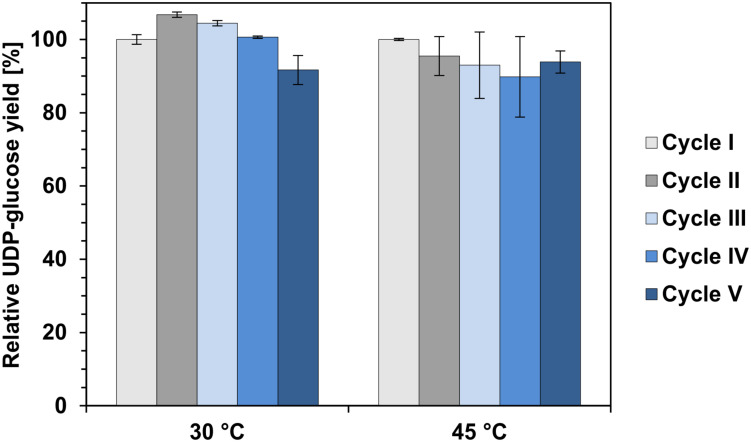
Repeatability in batch reaction with *Ta*GalU immobilized on MCF. Reaction mixture contained 2 mM UTP, 2 mM G1P, 3 mM MgCl_2_, 50 mM Hepes, pH 7.0, 0.3109 μg mL^–1^ *Ta*GalU immobilized amino modified MCF. Reaction was carried out at 30 and 45°C in 10 mL scale. Means with standard deviations are shown from duplicate reactions. The UDP-glucose yield after first cycle was set to 100%.

The main advantage of enzyme immobilization is the ease separation from the reaction solution and reusability. Therefore, the recyclability of *Ta*GalU immobilized on MCF in a batch system was investigated for five consecutive reactions (Figure 9). The reactions were carried out at two temperatures: 30 and 45°C and one cycle lasted for 5 min. These conditions were chosen according to the previous enzyme characterization as well as to ease of handling. After each cycle, the immobilized enzyme was centrifuged, washed with 50 mM Hepes buffer pH 7.0 and reused. Figure 9 shows that UDP-glucose production by immobilized *Ta*GalU maintained quite stable for at least five repeated cycles at both examined temperatures. Slight deviations in activity could be ascribed to the loss of carriers during washing in the repeated uses.

## Discussion

It was possible to produce and purify *Ta*GalU in reasonable amounts and with the second highest activity measured among UDP-glucose pyrophosphorylases from bacterial origin, to the best of our knowledge. The highest ever measured specific activity for a bacterial GalU was 5⋅10^6^ U mg^–1^ from the Firmicute *Streptococcus pneumonia* (Zavala et al., 2017). Activities from other bacterial GalUs are known to be much lower, like the one from *E. coli* K-12 with a *V*_max_ of 340 U mg^–1^ (Ebrecht et al., 2015) or the one from *Mycobacterium tuberculosis* H37Rv which had specific activities between 0.12 U mg^–1^ (Lai et al., 2008) and 2.7 U mg^–1^ (Asención Diez et al., 2015). Similar to ours, but still lower activities are only known from eukaryotic GalUs, like *Leishmania major* with 1477 U mg^–1^ (Steiner et al., 2007) or *Sorghum vulgare* with 1200 U mg^–1^ (Gustafson and Gander, 1972).

Since it was shown before that GalUs not only accept MgCl_2_ as a cofactor (Koo et al., 2014; Kumpf et al., 2019), we wanted to know, if the GalU enzyme from *Thermocrispum agreste* also accepts other metal ions. Here, we demonstrated the definite dependency of the enzyme activity on magnesium ions, but also that other divalent cations can be used for the reaction, like manganese, cobalt or zinc. The use of EDTA allowed to extract the divalent cations from the enzyme and thus lowered *Ta*GalU activity reinforcing the observation on magnesium dependency. Actually, the need of 50 mM EDTA to significantly lower the *Ta*GalU activity demonstrates how strong the binding of the magnesium ions to the active site of the enzyme is.

The specific activity of the free *Ta*GalU was shown to be very high, but the stability at elevated temperatures was relatively low. A short operational stability, recovery-reusability issues and shelf life often obstruct industrial application of free enzymes. To overcome these drawbacks, we studied a covalent immobilization of *Ta*GalU on to MCF. Based on our current state of knowledge there are only a few reports concerning UDP-glucose pyrophosphorylase immobilization (Haynie and Whitesides, 1990; Liu et al., 2002).

In our study, *Ta*GalU was covalently immobilized on MCF functionalized with amino or epoxy groups, which interact indirectly (using GA as a spacer) or directly with the functional groups of the enzyme. The immobilization was carried out at the pH range of 5.3–7.8, as the buffer pH is known to affect the enzyme structure as well as the reactivity of the functional groups of the carriers (Peng et al., 2012; Kim and Lee, 2019). It was generally observed that *Ta*GalU immobilized on amino-functionalized carriers showed much better activities than when immobilized on epoxy-modified silica. The highest specific activity was observed for *Ta*GalU immobilized on amino-modified MCF using phosphate buffer, pH 6.7. From the industrial/practical point of view, not only the activity of the biocatalyst is a very important factor, but also its stability. For this reason, the activity was measured after 1-month of storage at 4°C. All biocatalysts showed about 40% of their initial activity. For this reason, we selected a biocatalyst based on amino-modified MCF, pH 6.7, for further research.

It is believed that covalent immobilization is associated with a decrease in enzyme-specific activity, mainly due to conformational changes and decreased accessibility of active sites in the protein structure (Zhang et al., 2013; Karav et al., 2017). However, in our case, the specific activity of immobilized *Ta*GalU was 40% higher than the activity of the free form of the enzyme. This could be explained by the fact that an application of a spacer arm could provide higher mobility to the enzyme as well as minimize unfavorable steric hindrance caused by solid supports (Wang and Hsieh, 2004; Zhang et al., 2013; Karav et al., 2017). Thus, immobilization could change the enzyme configuration in a positive mode and along the activity, or during the process of immobilization the enzyme gets more enriched by a subsequent protein purification as properly folded proteins may bind more efficient to the carrier material as others which are likely still in the applied protein fraction (Jarzȩbski et al., 2007; Szymańska et al., 2007, 2013). The later might actually be the case as we observed the formation of inclusion bodies during protein production and so some slightly misfolded proteins might still be present but not easily separable.

It was also found that the immobilization of *Ta*GalU provided a broader temperature and pH profile compared to that of the free enzyme, which means that immobilization stabilizes the enzyme structure and thus maintain the enzyme activity in a wider range. Similar results were observed by other groups (Gahlout et al., 2017; Hassan et al., 2019). Various authors have suggested that it could be caused by the changes in physical and chemical properties of an immobilized enzyme. The covalent bond formation *via* amino groups might reduce the conformational flexibility, resulting in a higher activation energy for the molecule to reorganize the proper conformation of the binding substrate (Mazlan and Hanifah, 2017). On the other hand, the optimum pH for immobilized *Ta*GalU was shifted from 8.5 for the free enzyme to pH 7.5 after immobilization. The pH profile shift could be attributed to the fact that the microenvironment around the active site of the immobilized enzyme and the bulk solution usually has unequal partitioning of H^+^ and OH^–^ concentrations due to electrostatic interactions with the carrier (Mazlan and Hanifah, 2017; Zusfahair et al., 2017). Considering the long-term usage of biocatalysts, additional thermal stability tests for free and immobilized *Ta*GalU were made. For all tested temperatures, the immobilized enzyme showed a much better long-term stability than the free *Ta*GalU. Finally, at a temperature of 45°C, a decrease in the activity of immobilized *Ta*GalU was not observed, whereas the free enzyme lost more than 85% of its activity after 120 min. The structural rigidity of a covalently immobilized enzyme probably limits denaturation and inactivation upon heating (Ibrahim et al., 2016; Kim and Lee, 2019).

These features – a broader pH and temperature profile and thermal stability – are favorable in industrial applications. However, the most important advantage of enzyme immobilization is reusability (Daglioglu and Zihnioglu, 2012). The covalent binding method for enzyme immobilization provides a stronger bond compared to physical adsorption or the entrapment method; hence, an immobilized enzyme could be reused efficiently (Kim and Lee, 2019). In our case, immobilized *Ta*GalU was reused for five consecutive cycles without significant loss of activity.

The kinetic parameters were determined for both substrates: UTP and G1P. In the first case, UTP concentration was varied from 0.05 to 6 mM, keeping the G1P concentration constant at 30 mM. For G1P, the concentration was adjusted from 0.01 to 45 mM, holding UTP concentration at a value of 2 mM. The reaction was also performed without the addition of one of the substrates and as expected, no activity was detectable. The kinetic parameters of free *Ta*GalU were determined immediately after enzyme isolation (1016 U mg^–1^). Enzyme which was stored for about 1.5 years at 4°C remained a specific activity of 66 U mg^–1^, which represents 6.5% of the initial activity. This enzyme was still useful and applied in immobilization experiments. *K*_m_ and *K*_i_, which are the representative parameters for an enzymatic reaction and are not affected by the concentration or purity of the enzyme, can be compared straight on. It was observed that the power of the inhibitor measured by *K*_i_ constant was the same for the free and immobilized enzyme (see Supplementary Table 1). Whereas *Ta*GalU affinity toward substrates, determined by *K*_m_, were depended on the chemical structure of the substrate. The smaller molecule of G1P showed a slightly higher affinity (lower *K*_m_) to the free enzyme than UTP. For the immobilized *Ta*GalU the situation is *vice versa* with a lower affinity (higher *K*_m_) for G1P. In general, the affinity of the enzymes toward their substrates is lower (higher *K*_m_) for the immobilized *Ta*GalU than for the free form. This may be caused by the supported steric hindrance of the active site, by the change of enzyme flexibility after immobilization, or by diffusional resistance to the substrate transport. Additionally, UTP, as a reagent with a higher amount of phosphate groups, could strongly interact with the functional groups of the silica carrier or silica itself, limiting their accessibility to the enzyme (Keerti et al., 2014; Rahimizadeh et al., 2016). With the herein used experimental setup a strong substrate inhibition toward UTP could be detected, but no inhibition with higher concentrations of G1P. This indicates that the ratio between UTP and MgCl_2_ could be more important for the enzymatic reaction than concentration of G1P. It was also presented before that the complex of UTP and MgCl_2_ could be the actual substrate for the GalU reaction (Kleczkowski, 1994). Thus, it is suggested if both UTP and MgCl_2_ are present in solution that they form a transient complex which is readily bound by the enzyme and facilitating the reaction over the case in which both UTP and MgCl_2_ bind independently to the enzyme. This could explain why the concentration of UTP needs to be at least 10 times higher to detect a very small *Ta*GalU activity, than the concentration of G1P (see Figure 8). The ratio between UTP and MgCl_2_ (2 and 3 mM, respectively) was more optimal for the enzyme to catalyze the reaction when different G1P concentrations were tested. When the ratio between UTP and MgCl_2_ does not reach a certain threshold (in our case 0.1 mM UTP and 3 mM MgCl_2_), no activity for *Ta*GalU was measurable. In addition, it was reported that pre-incubation of the enzyme with UTP showed an increment in activity because of possible conformational changes of the GalU, which stabilizes the oligomerization of the GalU (Bosco et al., 2009). We also tested this hypothesis with both substrates and the free *Ta*GalU, but the activity was not increased.

## Conclusion

*Ta*GalU was successfully, covalently immobilized on MCF functionalized with amino groups after activation by glutaraldehyde. Immobilization enhanced the catalytic properties of *Ta*GalU. Furthermore, *Ta*GalU became more resistant to changes in temperature and pH. Moreover, it exhibited improved thermal long-term stability and reusability. These results confirm the economic and biotechnological benefits of enzyme immobilization and make *Ta*GalU an industrial interesting biocatalyst for the production of UDP-glucose. Further studies could also apply the immobilized *Ta*GalU in continuous processes as part of an enzyme cascade.

## Data Availability Statement

The datasets presented in this study can be found in online repositories. The names of the repository/repositories and accession number(s) can be found below: https://www.ncbi.nlm.nih.gov/genbank/, MT321102.

## Author Contributions

AK, DT, KS, IB, and AJ contributed conception and design of the study. AK did the majority of the experiments with the free enzyme. DK immobilized the enzyme and did the majority of the experiments with the immobilized enzyme. MM did parts of the experiments with the free enzyme. AŁ did parts of the immobilization experiments. AK and AP established the analytics for this study. AK and DK analyzed the data. KS and DT contributed to data analysis. AK wrote the first draft of the manuscript and visualized the data. DK, KS, and DT wrote sections of the manuscript and contributed to data visualization. All the authors contributed to manuscript revision, read and approved the submitted version.

## Conflict of Interest

The authors declare that the research was conducted in the absence of any commercial or financial relationships that could be construed as a potential conflict of interest.
