# Supplementary material for: Immobilization of the Highly Active UDP-Glucose Pyrophosphorylase From Thermocrispum agreste Provides a Highly Efficient Biocatalyst for the Production of UDP-Glucose
Source: Front Bioeng Biotechnol. 2020 Jul 2;8:740. doi: 10.3389/fbioe.2020.00740 (PMC7343719; doi:10.3389/fbioe.2020.00740)
Supplement: Supplementary file 1 [file Data_Sheet_1.pdf]

Supplementary Material

Immobilization of the highly active UDP-glucose pyrophosphorylase from *Thermocrispum agreste* provides a highly efficient biocatalyst for the production of UDP-glucose

Antje Kumpf[1,2,3,*], Daria Kowalczykiewicz[4,5], Katarzyna Szymańska[6], Maria Mehnert[1], Isabel Bento[3], Aleksandra Łochowicz[4], André Pollender[1], Andrzej Jarzębski[6,7], Dirk Tischler[2,*]

[1] Institute of Biosciences, Environmental Microbiology, TU Bergakademie Freiberg, Freiberg, Germany

[2] Microbial Biotechnology, Faculty of Biology and Biotechnology, Ruhr-Universität Bochum, Bochum, Germany

[3] EMBL Hamburg, Hamburg, Germany

[4] Department of Organic Chemistry, Bioorganic Chemistry and Biotechnology, Silesian University of Technology, Gliwice, Poland

[5] Biotechnology Center, Silesian University of Technology, Gliwice, Poland

[6] Department of Chemical and Process Engineering, Silesian University of Technology, Gliwice, Poland

[7] Institute of Chemical Engineering, Polish Academy of Sciences, Gliwice Poland

*** Correspondence:**
Antje Kumpf
antje.kumpf@rub.de

Dirk Tischler
dirk.tischler@rub.de

Keywords: *Thermocrispum agreste* DSM 44070, NDP-sugars, UTP, immobilization, silica carrier, mesostructured cellular foams, MCF

The supplemental material comprises the following information; codon optimized gene encoding the employed biocatalyst as well as activity data regarding divalent cations, buffers, potential inhibitors, solvents and kinetic data describing soluble and immobilized enzyme preparations.

The *Ta*GalU gene (accession number: WP_028847555) was codon optimized (accession number: MT321102) and synthesized prior cloning.

>codon optimized gene plus restrictions sites for cloning (NdeI and NotI; underlined)

CAT ATG ACG GCG AGT GCC GAG CAT CAG ACC TTC ACC ACT GCA ATT GTG CCT
GCA GCT GGC CTC GGT ACG CGC TTT CTG CCC ACC ACC AAA TCG GTG CCG AAG
GAA TTG CTG CCT GTC GTG GAT ACA CCG GCG ATT GAG CTT GTG GCG GAC GAA
GCG CGT CAA GCG GGC GCG GAA CGT CTG GTG ATC GTG ACG TCA CCA GCT AAA
CAG AGC ATT GCT GCC TAC TTT CGC CCA GCT CCG GAA CTG GAA CGC TCG TTG
GAA GAG AAG GGC AAA ACC GGA CAA CTC GCG AAA ATT CGC CGT GCA CCA GAA
CTG CTT GAG GTA GAG GTT GCG ATC CAG GAA CAG GCT CTG GGG TTA GGT CAC
GCA GTT GCC TGT GCC GAA CCT AAC CTG GGT CCG GAA GAT GAC GTA GTT GCC
GTT CTG CTG CCG GAT GAT CTG GTC CTT CCA CAC GGC ATC CTG GAA CGC ATG
GCG AAA GTT CGT GCC GAA CAT GGC GGC TCT GTG CTC TGC GCG TTT GAC ATC
CCG AAA GAG GAA ATT AGC GCG TAT GGG GTT TTC GAT GTG AGC GAC ACG GAT
GAT GCG GAC GTG AAA CGG GTG CAC GGT ATG GTC GAG AAA CCG CCT GCA GAA
CAA GCA CCC TCC ACT TTT GCC GCC GCA GGC CGT TAC TTG CTG GAT CGT GCG
ATC TTC GAT GCG TTA CGT CGC ATT GAA CCC GGT GCG GGT GGA GAG CTG CAG
CTG ACA GAT GCC GTC GCT TTG CTG ATT CAG GAA GGA CAT CCG GTA CAC GTC
GTC GTA CAT CGC GGT GAT CGG CAT GAC CTC GGC AAT CCG GGT GGG TTC CTG
CGC GCT GCA GTT GAC TTT GCA CTG CAG GAT CCG GAC TAT GGC CCG GAA TTA
CGC GCC TGG TTA ACC GAT CGC ATT GCC CGT CCG TGA GCG GCC GC

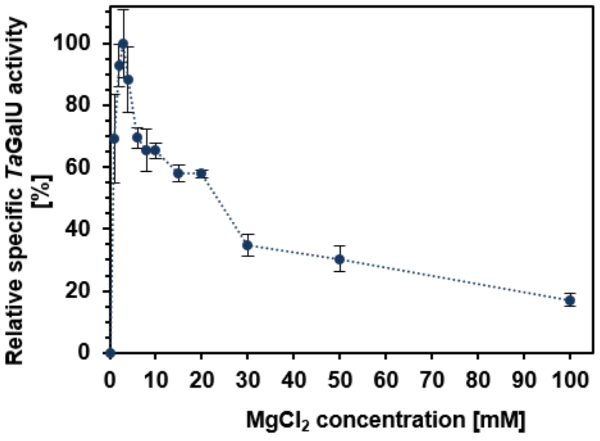

Supplementary Figure 1. Relative specific *Ta*GalU activity depending on the applied magnesium chloride concentration. Reaction solution contained 2 mM UTP, 2 mM G1P, 0-100 mM $MgCl_2$, 50 mM Hepes, pH 7.0, 0.013 µg mL^{-1}, 50 °C, 1 mL reaction volume. 100 % relative activity corresponds to 2111 U mg^{-1}. Means with standard deviations of triplicate measurements are shown.

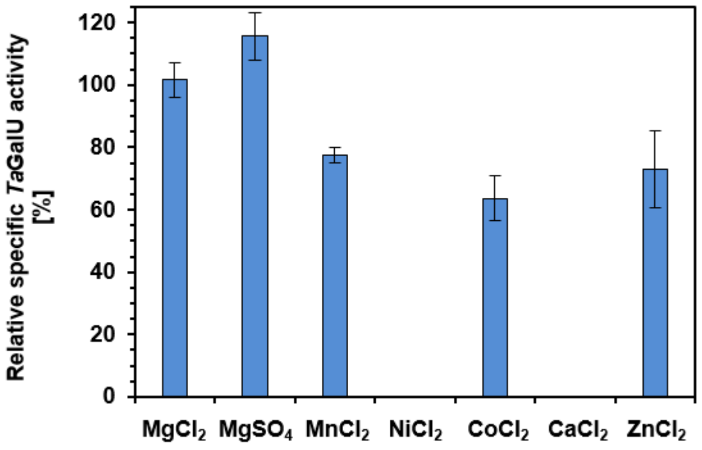

Supplementary Figure 2. Different metals as cofactors tested in the *Ta*GalU reaction. 1 mL reaction solution contained 2 mM UTP, 30 mM G1P, 3 mM metal salt, 50 mM Hepes, pH 7.0 and 0.013 µg mL^{-1} *Ta*GalU. Reaction was carried out at 50 °C. 100 % corresponds to 1256 U mg^{-1}. Means with standard deviations of triplicate measurements are shown.

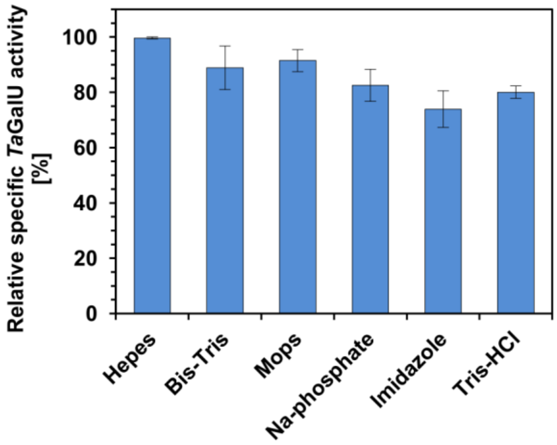

Supplementary Figure 3. Different buffers tested for the *Ta*GalU reaction. 1 mL of reaction mixture contains 2 mM UTP, 30 mM G1P, 3 mM $MgCl_2$, 50 mM buffer, pH 7.0 and 0.013 µg mL^{-1} *Ta*GalU. The reaction temperature was 50 °C. 100 % corresponds to 1258 U mg^{-1}. Means with standard deviations of triplicate measurements are shown.

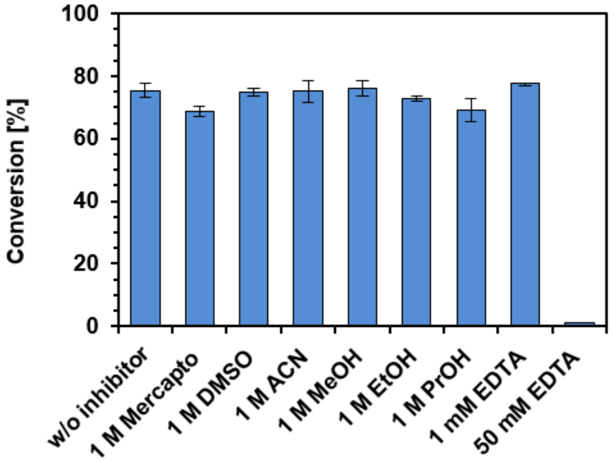

Supplementary Figure 4. Effect of inhibitors or solvents to *Ta*GalU. The reaction mixture contained: 2 mM UTP, 30 mM G1P, 3 mM $MgCl_2$, 50 mM Hepes, pH 7.0, 1 mM and 50 mM EDTA, respectively or 1 M solvent/inhibitor, 0.013 µg mL^{-1} *Ta*GalU. Reaction was carried out at 50 °C in 200 µL reaction volume. Means with standard deviations are shown. Mercapto: 2-mercaptoethanol, DMSO: dimethyl sulfoxide, ACN: acetonitrile, MeOH: methanol, EtOH: ethanol, PrOH: isopropanol, EDTA: ethylenediaminetetraacetic acid.

Supplementary Table 1. Kinetic constants of the free and immobilized *Ta*GalU calculated by fits

kinetic constant	Free		Immobilized		Immobilized and multiplied by factor	
	UTP	G1P	UTP	G1P	UTP	G1P
K_m [mM]	0.15	0.12	0.40	0.79	0.40	0.79
V_{max} [U mg^{-1}]	1698	1109	226	157	3480[a]	2418[a]
k_{cat} [s^{-1}]	914	597	121	84	1874[a]	1302[a]
K_I [mM]	3.9	-	3.9	-	3.9	-
k_{cat}/K_m [μM^{-1} s^{-1}]	6.09	4.98	0.3	0.11	4.69[a]	1.65[a]

[a]: For data collection of the kinetics for the immobilized *Ta*GalU an old batch of enzyme with reduced specific activity was used. Therefore, we recalculated the values for V_{max} with the time factor 15.4. This factor emerged from the fact that the initial specific activity of the free enzyme was 1016 U mg^{-1} directly after purification. After storage of the enzyme for 1.5 years the activity decreased to only 66.03 U mg^{-1}, which is a reduction by the factor 15.4. Therefore, these values are of theoretical nature.
